# Association between Sleep Disturbances and Leisure Activities in the Elderly: A Comparison between Men and Women

**DOI:** 10.1155/2014/595208

**Published:** 2014-01-19

**Authors:** Amanda Hellström, Patrik Hellström, Ania Willman, Cecilia Fagerström

**Affiliations:** ^1^School of Health Science, Blekinge Institute of Technology, 371 79 Karlskrona, Sweden; ^2^Department of Health Sciences, Lund University, 221 00 Lund, Sweden; ^3^Department of Care Science, Malmö University, 205 06 Malmö, Sweden; ^4^Blekinge Centre of Competence, 371 81 Karlskrona, Sweden

## Abstract

It has been suggested that physical or social activity is associated with fewer sleep disturbances among elderly people. Women report more sleep disturbances than men, which could indicate a variation in activity patterns between the genders. The aim of this study was to investigate associations between sleep disturbances and leisure activities in men and women (*n* = 945) aged ≥60 years in a Swedish population. Sleep disturbances were measured using eight dichotomous questions and seventeen variables, covering a wide range of leisure activities. Few leisure activities were found to be associated with sleep disturbances and their importance decreased when the models were adjusted for confounders and gender interactions. After clustering the leisure activities and investigating individual activities, sociointellectual activities were shown to be significant for sleep. However, following adjustment for confounders and gender interactions, home maintenance was the only activity significant for sleep. Being a female increased the effect of home maintenance. Besides those leisure activities, poor/fair self-rated health (OR 7.50, CI: 4.27–11.81) and being female (OR 4.86, CI: 2.75–8.61) were found to have the highest association with poor sleep. Leisure activities pursued by elderly people should focus on activities of a sociointellectual nature, especially among women, to promote sleep.

## 1. Introduction

It is suggested that leisure activities, such as physical and social activities and spending time outdoors, influence the timing of sleep and the robustness of the sleep-wake rhythm [1, 2]. Physical and social activities have also been found to improve sleep quality, efficiency, and duration [3, 4]. However, extremely short/long sleep duration has been associated with higher morbidity among elderly people (≥60 years) [5]. Fewer daytime activities and frequent naps during the day contribute to changes in the sleep-wake rhythm, which might lead to poor sleep quality [6]. This implies that elderly people need stimulating activities during the day if they are to sleep well at night.

Sleep changes throughout life. Elderly people usually go to sleep and rise earlier (i.e., phase-advanced sleep) [1], especially elderly women [7]. There is a decrease in sleep efficiency and deep sleep (N3) [6]. The most common sleep disturbances in elderly people are nocturnal awakening, difficulty falling asleep, and early awakening [8]. Some of the changes in sleep can be attributed to normal ageing and others to medical conditions [1]. Most of the insomnia that occurs in old age is chronic, and long-term use of sleep medication is known to lead to impaired memory and daytime functioning [8]. There is thus a need for nonpharmacological management of sleep disturbances among elderly people.

Several randomised controlled trials (RCTs) [3, 9–11] have demonstrated sleep benefits when performing activities in people aged ≥55 years. Low-intensity physical activities, such as tai-chi, as well as high-intensity physical activities and social activities, all improve sleep quality, duration, and efficiency [3, 9, 10]. However, instead of investigating a set of predetermined activities, an alternative approach could be to investigate the activities that elderly people pursue in their leisure time. Leisure activities can be defined as activities that are enjoyable for the individual, are chosen freely, and derive from the interests and skills of the individual [12]. Leisure activities can include physical, social as well as cultural, or creative components.

Activity has been defined with considerable variation. It cannot be concluded therefore that all activities influence a common physiological mechanism, or that activities affecting sleep depend on energy expenditure. Morgan [13] investigated different levels of customary physical activity (CPA) and sleep quality. Included in CPA were outdoor productive activities (e.g., gardening), indoor productive activities (e.g., housework), outdoor walking, shopping, and leisure activities. The results showed that CPA was related significantly to insomnia whereas social engagement and daily walks were not [13]. Associations between physical and/or social activities and sleep have been investigated previously in several studies although the findings are inconclusive regarding the establishment of statistically significant improvements in sleep. Another difficulty is the definition of “physical and social activity”. The inconclusive findings could possibly be explained by the nature of the activities that were included or that they are sorted into physical or social. Not investigating activities solely on the group level but also as individual activities could lead to greater understanding of which activities might be beneficial to sleep.

In elderly people, the association between leisure activities and sleep disturbances cannot be investigated without taking into account confounding variables. A confounder can be described as a variable that blurs or distorts the real effect of exposure [14]. Possible confounders of the association between sleep disturbances and leisure activity are gender, cognition, health, functional ability, and mood. Sleep disturbances in elderly people are found to be associated with female gender, depression, impaired cognitive function, and perceived poor health [15–18]. The association between poor health and sleep disturbances in people aged ≥60 years is well known [5, 19, 20] although sleep may also be affected by the functional status of the person [7]. In order to be active, a person needs functional ability and a willingness to engage in activities. Sleep disturbances have also been linked to mood disorders and other psychiatric illnesses among elderly people [21, 22]. Furthermore, mood disorders, such as depression or rumination and neuroticism, are more frequent in women [23].

Women report sleep disturbances to a greater extent [24], and sleep disturbances seem to increase with old age [25] as does the use of sleep medication [26]. Investigation of active ageing in the elderly showed that women are more often widowed, have a lower level of education, and are homemakers. Women also report more diseases and poorer self-rated health compared to men [27]. The different prerequisites of men and women could imply that men and women also have deviating activity patterns. Previous research highlights the seriousness of sleep disturbances and the importance of investigating sleep-promoting interventions in men and women separately. The aim of this study was to investigate associations between sleep disturbances and leisure activities in elderly (≥60 years) men and women in a Swedish population.

## 2. Methods

### 2.1. Design and Sample

This cross-sectional study included participants (*n* = 945) enrolled in the longitudinal “Swedish National Study on Aging and Care-Blekinge” (SNAC-B). Data were provided by the survey, which was carried out in 2007–2009. Participants lived in a municipal area in south-east Sweden with a population of approximately 64,000. Ten age clusters, ranging from 60 to 96+ years, were included in the SNAC-B sample. There was randomised selection of the four younger age clusters (60, 66, 72, and 78 years) and total inclusion of the clusters of people aged 81, 84, 87, 90, 93, and 96+ years [28]. The SNAC-B sample is intended to be representative of the Swedish population. However, in the present study there was a slight overrepresentation of the oldest age (90+). Data within the SNAC-B study were collected through medical examinations, interviews, and self-administered questionnaires [28]. Demographic data, as well as data on sleep, leisure activities, functional status, and general health, were collected from the SNAC-B self-administered questionnaire.

### 2.2. Measurements

Sleep disturbances were measured by means of eight questions developed for the Comprehensive Assessment and Referral Evaluation interview schedule [29] and which were applied previously in a three-year followup study of elderly people [30]. The questions are dichotomous (yes/no) and based on self-reports. Examples of questions are as follows. *Is your sleep interrupted during the night?*, *Do you have difficulty falling asleep?,* and *Do you wake up early?* A cut-off figure of ≥4 sleep disturbances at the third quartile of the total sample was considered to be the demarcation point between those who sleep well (0–3 sleep disturbances) and those who sleep poorly (≥4 sleep disturbances). The cut-off point is based on the assumption that 10–25% of the general population suffer from persistent insomnia and with a higher prevalence among people aged >65 years [31, 32]. For the descriptive analyses, questions about use of sleep medication and sleep duration were added.

Leisure activities were measured using 17 dichotomous items, covering a wide range of activities (Table 1). In order to obtain a picture of the participants' exercise habits, two questions concerning light and intensive physical activity were amalgamated into one variable and dichotomised question, revealing whether or not the person performed regular exercise. The new variable comprised activities such as jogging, walking, cycling, gardening, exercising, swimming, skiing, skating, and ball games. The highest frequency was registered, irrespective of intensity. All people who participated in physical activity at least once a week were considered to exercise. Leisure activities were investigated as clusters of activities and as individual activities (Table 1).

Functional status was measured using the Instrumental Activities of Daily Living (IADL) items: shopping, cooking, cleaning, washing, and transportation. Each item had the following response alternatives: dependent, partly dependent, or independent. The people who were dependent and partly dependent were classified as dependent, thus creating five dichotomous IADL items. The five items were then amalgamated into a dichotomous variable (dependent on one or more activity/independent).

Mood was measured by using a subscale of the Life Satisfaction Index [33]. In a factor analysis by Liang [34], three factors were found: mood, zest, and congruence. These factors have been validated in a sample of elderly people [35]. The mood factor comprises three items:* I am just as happy as when I was younger, My life could be happier than it is now,* and* These are the best years of my life. *All questions are answered with agree, uncertain, or disagree. The item *My life could be happier than it is now* was reversed before the total score was computed. The response alternatives “uncertain” and “disagree” were amalgamated for items 1 and 3, and “agree” and “uncertain” were amalgamated for *My life could be happier than it is now*. Mood scores are shown as being above or below the median value of the measurement (Md 1, range 0–3).

General health was measured using an item from the Short Form 12 questionnaire (SF12) with five response alternatives, ranging from poor to excellent [36]. The five original responses were then transformed into three responses: poor/fair health, good health, and very good/excellent health. This was done in order to fit the variable's variation in the sample.

Cognition was measured using the *Mini Mental State Examination* (MMSE), which measures various cognitive processes. It can be used as a screening device for cognitive impairment, and three levels of cognition are defined. The score range is 0–30, where a score of 0–17 indicates severe cognitive impairment, 18–23 indicates mild cognitive impairment, and 24–30 indicates no cognitive impairment [37]. For this study, a cut-off at ≥24 of the MMSE has been made, separating those with cognitive impairment, regardless of severity, from those without impairment.

### 2.3. Statistical Analysis

Data were analysed using PASW Statistics 21.0 (SPSS Inc. Chicago, IL, USA). Descriptive analyses and group comparisons were made using the *χ* ^2^ test and the Mann-Whitney *U*-test on continuous variables. Yates' continuity correction was used in four field tables [38]. The ten age clusters used in SNAC-B were reduced to three clusters: 60 and 66 years (retirement age); 72- and 78-year-olds; and 81 years and above, in order to increase the number of people in each cluster.

Associations between variables were calculated using Spearman's RHO and multiple logistic regressions. Correlations using Spearman's RHO between sleep disturbances, confounding variables, and leisure activities were investigated. Only variables found to be significant (*P* < 0.05) through crosstabulation and associated with sleep disturbances in Spearman's RHO (Table 1) were entered into the multiple logistic regressions. The modelling was performed in three steps. Firstly, associations between sleep disturbances and clustered activities were investigated. Secondly, associations between sleep disturbances and individual leisure activities were investigated. Thirdly, associations between sleep disturbances and individual leisure activities and interactions between gender and the main effects found in the second model (2a) were investigated. All the crude models were adjusted for the confounder variables: gender, age, general health, functional ability, and mood. All multiple logistic regressions were performed using a backward, stepwise likelihood ratio (LR) method and were presented as odds ratios (ORs) with 95% confidence intervals (CIs). Goodness of fit of the regression models was performed using the Hosmer-Lemeshow test [39]. Collinearity diagnostics (VIF) was used to check for multicollinearity between the independent variables and was shown to be acceptable. Response alternatives that were considered to have the lowest association with ≥4 sleep disturbances were chosen as references for each variable. Subjects with an internal dropout in one or several variables were excluded from the models. The level of significance was set at *P* < 0.05.

### 2.4. Ethical Considerations

Written and verbal consent was obtained from participants. The study was approved by the Regional Ethical Review Board in Lund (LU 605-00, LU 744-00).

## 3. Results

### 3.1. Sample Description

Of the 945 people included in the study, 55.4% were women. The mean age of the women was 74.3 years (SD 10.3), while the mean age of the men was 72.8 years (SD 10.1) (Table 2). Women reported greater use of sleep, medication, greater dependence on medication for sleep, and more coexisting sleep disturbances than men (Table 2). Sleep disturbances due to pain and itching tended to be more common in women. The mean sleep duration was 6.7 hours for women and 6.9 hours for men. Sleep duration in the sample ranged from two to 12 hours, representing extreme sleep durations. Although there was a significant difference between the genders, both men and women reported interrupted sleep during the night, waking up early, and difficulty falling asleep as the three most common sleep disturbances. There were no significant differences between the genders with regard to early waking and daytime napping (Table 2).

There were significant differences in sleep disturbances between age cohorts and with regard to general health, functional status, and mood. Among the poor sleepers, 59.8% perceived their general health as poor or fair compared to 27.4% of the good sleepers. People with poor sleep were frequently more dependent when performing daily activities than those who slept well (34.5% versus 16.6%). Of those who slept poorly, 70.6% reported low mood (Table 3).

### 3.2. Sleep Disturbances and Leisure Activities

The most common leisure activities among both poor and good sleepers were reading the daily paper (95.5% versus 97.7%), reading journals or magazines (84.5% versus 86.4%), watching television (95.9% versus 97%), and listening to music (87.7% versus 89.4%) (Table 3). Significant differences existed between poor and good sleepers with regard to performance of regular exercise, gardening, strolling in the country, picking berries, home maintenance, repairing cars/machinery, playing chess/cards, and using the Internet/playing computer games.

Logistic regressions were performed to assess the associations between leisure activities and reports of ≥4 sleep disturbances. The first model contained the two clusters of leisure activities found to be associated significantly with sleep disturbance using univariate analyses (Table 1). Performing no sociointellectual activities increased the odds ratio of sleep disturbances (OR 3.75, CI: 1.49–9.43) as did performing only one sociointellectual activity (OR 3.15, CI: 1.26–7.88) compared to performing four sociointellectual activities. Physical outdoor activities did not contribute to Model 1a (Table 4). After adding the confounding variables gender, age cohorts, functional ability, general health, and mood (Model 1b, Table 4), none of the clusters of leisure activities mattered. Instead, being a woman (OR 3.12, CI: 2.16–4.52), aged 81 or older (OR 1.68, CI: 1.09–2.58), and perceiving one's health as good (OR 3.36, CI: 2.00–5.62) or poor/fair (OR 6.97, CI: 4.24–11.45) increased the odds ratio (OR) of having poor sleep.

A second model was then performed: investigating associations between sleep disturbances, individual leisure activities, and confounding variables (Table 5). Five leisure activities were found to be the main effects. Gardening (OR 1.45, CI: 1.00–2.08), strolling in the country (OR 1.47, CI: 1.01–2.14), home maintenance (OR 1.60, CI: 1.08–2.39), repairing cars/machines (OR 2.20, CI: 1.28–3.77), and playing chess/cards (OR 1.69, CI: 1.13–2.52) were all found to be significant for poor sleep (Model 2a). However, after adjusting for health, functional ability, gender, mood, and age, the highest odds ratio of sleep disturbances were among those reporting poor/fair health (OR 6.82, CI: 4.14–11.22), followed by those with good health (OR 3.33, CI: 1.99–5.58). Being a woman compared to being a man yielded three times the odds of poor sleep (OR 3.06, CI: 2.11–4.43). People aged 81 or older were 1.60 times likely to report poor sleep compared to those of retirement age. No significant difference was found between those of retiring age and those aged 72 or 78 years (OR 0.91, CI: 0.59–1.41). Of the leisure activities, only playing chess/cards, which is a sociointellectual activity, remained significant, with an odds ratio of 1.54 (CI: 1.01–2.35) (Table 5).

The second part of the aim was to investigate differences between men and women with regard to leisure activities associated with sleep disturbances and interactions between gender and activities. A third model was created containing the main effects found in Model 2a (gardening, strolling in the country, home maintenance, repairing cars/machines, and playing chess/cards). “Gender” was also added to the equation, as well as interactions between gender and each of the activities. Performing home maintenance (OR 2.26, CI: 1.17–4.35), playing chess/cards (OR 1.61, CI: 1.09–2.39), strolling in the country (OR 1.56, CI: 1.07–2.29), and gardening (OR 1.50, CI: 1.04–2.16) were the leisure activities linked to sleep disturbances (Table 6). Examination of the interactions between gender and activities showed that the interaction between gender and home maintenance had an OR 0.44 (CI: 0.20–0.94). The interpretation is that being a man and being active did not increase the effect on sleep disturbances. After adjusting the model with confounder variables (Table 6, Model 3b), the home maintenance activity decreased in significance (OR 2.09, CI: 1.07–4.07), as did the interaction between gender and home maintenance (OR 0.33, CI: 0.15–0.75). Other explanatory variables for sleep were poor/fair health, good health, and being a woman, while the age clusters did not reach statistical significance (Table 6). People who reported poor/fair health were almost seven times more likely to report having ≥4 sleep disturbances, allowing for all the factors in the model.

## 4. Discussion

The associations between leisure activities and sleep disturbances were investigated as well as comparisons between men and women in an elderly Swedish population. The number of activities performed was of less importance than performing specific activities. Physical outdoor and socio-intellectual activities were those that were found to be of significance, both as clustered and individual activities. Individual activities presented as being associated significantly with fewer sleep disturbances were gardening, strolling in the country, playing chess/cards, repairing cars/machines, and home maintenance. Following modelling and the inclusion of confounders and gender interactions, it became clear that it is mainly sociointellectual activities that were of importance. Interactions between leisure activities and gender showed that home maintenance was significant in relation to sleep disturbances, especially in women. This remained after the model was adjusted.

Gardening and strolling are outdoor activities, implying spending time in daylight/sunlight, which is known to be beneficial for sleep. Bennett [40] found that women preferred to perform indoor leisure activities, such as housework, whereas men frequently did more gardening and car maintenance. Armstrong and Morgan [41] also found that women performed outdoor activities to a lesser degree than men. If women are less likely to be involved in outdoor activities, this could be a possible explanation for the higher prevalence of sleep disturbances among women. Environmental and social factors, such as being widowed or being a homemaker, are also associated with poorer sleep [24, 42]. These are circumstances that could have contributed to the observed gender differences but they were not investigated here and cannot be verified or refuted by the present study.

Playing chess/cards was defined as a sociointellectual activity that exercises the memory and mental activity and maintains social contacts and communication [43]. Playing games might have a possible effect on brain plasticity, which refers to physical changes in the neurons in response to stimuli. Motivating or challenging stimuli enhance connections between neurons in the brain, thus improving or maintaining cognitive ability [44]. Findings from human and animal studies show that the need for sleep is adjusted by the amount of brain plasticity during prior waking [45]. Exposure to challenging and novel experiences could possibly trigger homeostatic increases in sleep requirements and thus also in deep sleep [44, 45]. Chess in particular is said to stimulate memory, attention, concentration, creativity, and reasoning, which underlines the value of this activity in the elderly. Playing games requires rigorous thinking combined with agility [46]. Social interactions have also been found to protect against depression [47], which often occurs in conjunction with sleep disturbances [21].

Home maintenance was the only significant individual activity for sleep when the model was adjusted for confounding variables and gender interactions. Unfortunately, it is not feasible to verify the upcoming finding since the same association has not been described previously. However, home maintenance could provide intellectual stimulation and physical activity and be a marker of autonomy, depending on the task. In an Australian study, having your own home was considered to be a sign of being free and not having to answer to anyone. The house was seen as a symbol of independence and autonomy [48]. This is also supported by a study of remote communities in Scotland. Even if tasks in the home might take longer for an elderly person to perform, it was important to remain independent [49]. In both studies it was emphasised that staying in the house also meant keeping your social contacts [48, 49]. It is possible that home maintenance represents more than just the physical performance of maintenance.

It was interesting to note in our study that the interaction showed that being both male and active did not have a synergetic effect with regard to home maintenance. The addition of confounding variables to the model did not erase the effect of the interaction between home maintenance and gender. It remained significant but the odds were lower, that is, decreasing the effect of not being an active man. The gender difference could be explained by the high odds for gender (OR 3.63, CI: 2.15–6.15) and home maintenance itself (OR 2.26, CI: 1.17–4.35). The finding also implies that home maintenance is of greater importance to women than men with regard to sleep disturbances. Even if the findings cannot be validated by previous research, it is possible that the ability to maintain one's own household independently could be a marker that distinguishes poor sleepers from those with fewer sleep disturbances. Nevertheless, this needs to be investigated further.

Another factor affecting sleep was health. Perceiving one's general health as fair/poor stood out as the strongest variable associated with sleep disturbances, followed by gender and good general health in the final model (3b). Physical and mental factors, such as medical illnesses, low mood, physical disabilities, and poor perceived health, are known to be associated with sleep disturbances [42]. Women reported poor sleep to a greater degree than men (33.9% versus 13.9%). This is concordant with previous research, where women tend to have more difficulty sleeping than men [24], although studies that included objective measurements indicate the opposite [7]. Use of sleep medication was higher for women than for men (18.4% versus 9.4%), which confirms the findings of Ineke Neutel and Patten [26]. A possible interpretation of the results could be that women have difficulty sleeping despite the use of sleep medication. Prolonged use of sleep medication in women has been associated with perceived poor health and negative health outcomes [19]. It could be assumed that the higher prevalence of sleep disturbances in women was related to poor health. Another explanation could be that women did not sleep quite as long as men, and in the present study shorter sleep was found to be associated significantly with sleep disturbances. Women reported poor/fair health, low mood, and impaired physical ability to a greater extent than men. Tanaka and Shirakawa [50] suggest that sleep could be the key to improving or maintaining mental and physical health among the elderly, underlining the importance of sleep promotion, especially in women. There are obvious differences between the sleep of men and women, and tailored interventions for sleep promotion that take into account gender should be considered.

An advantage of the study was that an investigation was made of a broad spectrum of leisure activities that varied in intensity and orientation. Previous research emphasises that nonpharmacological interventions aimed at sleep hygiene factors may improve nocturnal sleep and maintain cognitive functioning and quality of life [32, 51]. Consequently, management of sleep disturbances by encouraging active living may result in several health benefits. No causalities can be inferred from the findings although it is known that sleep deprivation has a detrimental effect on brain function. Sleep loss could also result in a decrease in the restorative functions of the body as well as immune functions, which implies reduced resistance to infections [50].

The sample in this study is representative of the ageing population in Sweden, which is an advantage when investigating factors that affect sleep disturbances. The downside of the data collection procedure is that the most fragile elderly people do not have the strength or willingness to participate. This is mirrored in part by the large number (77.4%) of physically independent people with very good/excellent health and mood who were included in the study. The sample resided in what is mostly a sparsely populated area with small towns, which may have affected the leisure activities that were pursued. Geographical differences in the selection of leisure activities have been shown previously [52]. Other activities that were crucial to sleep could possibly have been found if the investigation was in a city area or in another part of Sweden. The study sample was taken from a single geographical area of Sweden, which implies a need for further studies. Interestingly, commonly performed activities, such as exercise or reading, did not explain the variation in sleep disturbances.

The decision to make a cutoff at the third quartile of the sample with regard to good and poor sleep was based on previous studies. Approximately 30–60% of the general population in developed countries suffer from insomnia symptoms [21, 53, 54], 10–20% of whom have persistent insomnia [31]. Among elderly people, 12–25% of those aged ≥65 years have persistent insomnia [32]. The prevalence varies depending on whether it is symptoms of insomnia or persistent insomnia that are measured. The use of eight single items with dichotomous answers when measuring sleep disturbances meant that only the number of difficulties could be measured, not the frequency, which is a common measurement in sleep studies. However, the questions picked up features of insomnia well, insomnia being one of the most common sleep disturbances. The questions were thus considered relevant.

## 5. Conclusions

Our findings show that sociointellectual activities are beneficial for sleep. Physical activities, such as strolling in the country or gardening, were significant in the crude models although they became nonsignificant when the models were adjusted. Including gender interactions, home maintenance was the only activity found to be significant for sleep, particularly in women. Furthermore, it was emphasised that people who perceived their health as poor/fair ran a greater risk of sleep disturbances. Self-rated health and the ability to manage your own home could be important markers for sleep disturbances. However, the significance of doing home maintenance cannot be validated by previous research and needs to be investigated further.

## Figures and Tables

**Table 1:** The leisure activities presented as clusters and individual activities and their association with sleep disturbances.

Cluster of leisure activities	Individual leisure activities	Correlation with sleep disturbances		*P* value	
		Group level	Individual level	Group level	Individual level
Physical outdoor activities		−0.223		**<0.001**	
	Exercise		−0.095		** 0.007**
	Gardening		−0.167		**<0.001**
	Strolling in the country		−0.174		**<0.001**
	Picking berries		−0.140		**<0.001**
	Hunting and fishing		−0.121		** 0.001**

Sociointellectual activities		−0.248		**<0.001**	
	Home maintenance		−0.215		**<0.001**
	Repairing cars/machines		−0.193		**<0.001**
	Playing chess/cards		−0.108		** 0.002**
	Using/surfing the Internet/playing computer games		−0.129		**<0.001**

Creative activities		0.047		0.165	
	Knitting, weaving, or sewing		−0.090		** 0.010**
	Playing an instrument		−0.040		0.286
	Painting, drawing, or pottery		−0.012		0.832

Cultural activities		−0.005		0.888	
	Reading a daily paper		−0.060		0.125
	Reading magazines/journals		−0.023		0.560
	Reading books		−0.014		0.751
	Watching TV		−0.026		0.578
	Listening to music		−0.024		0.562

Note: correlations between clusters and individual leisure activities were calculated using Spearman's RHO and Pearson's Chi-squared test.

**Table 2:** Sleep disturbances, sleep duration, sleep medication, general health, functional status, cognition, and mood among men and women in the total sample. Percentages in brackets, significant values in bold.

	Men *n* = 421	Women *n* = 524	*χ* ^2^ value	df	*P* value	Missing data (*n*)
Mean age (SD)	72.8 (SD 10.1)	74.3 (SD 10.3)			** 0.035** ^ 2^	0
Difficulty falling asleep	74 (17.9)	188 (36.5)	38.2	1	**<0.001** ^ 1^	17
Taking or being dependent on medication for sleep	51 (12.3)	103 (19.9)	9.3	1	** 0.002** ^ 1^	12
Sleep interrupted during the night	325 (77.9)	446 (86.4)	11.0	1	** 0.001** ^ 1^	12
Difficulty sleeping (falling/staying asleep) due to moods or tension	71 (17.3)	165 (32.7)	27.5	1	**<0.001** ^ 1^	30
Difficulty sleeping due to pain or itching	54 (13.0)	111 (21.8)	11.5	1	** 0.001** ^ 1^	21
Inability to return to sleep after waking at night	45 (10.9)	112 (21.8)	18.7	1	**<0.001** ^ 1^	17
Waking up early	236 (57.0)	308 (60.2)	0.8	1	0.367^1^	19
Feeling tired and sleeping for more than two hours during the day	33 (8.0)	39 (7.6)	0.0		0.910^1^	17
Sleep disturbances 0–8			45.9	1	**<0.001** ^ 1^	61
Poor sleep ≥4	55 (13.9)	165 (33.9)				
Good sleep 0–3	342 (86.1)	322 (66.1)				
Sleep duration in hours	6.9 (SD 1.2)	6.7 (SD 1.3)			** 0.048** ^ 2^	119
Mean, (SD), (min–max)	(3–12)	(2–11)				
			7.56		**0.023** ^ 3^	119
Short sleep ≤5 h	39 (10.9)	83 (17.7)				
Normal sleep 6–9 h	312 (87.2)	376 (80.3)				
Long sleep ≥10 h	7 (2.0)	9 (1.9)				
Prescribed sleep medication			20.9	4	**<0.001** ^ 3^	10
Never	339 (81.5)	367 (70.7)				
Sometimes per month	35 (8.4)	48 (9.2)				
Several times per month	3 (0.7)	9 (1.7)				
Sometimes per week	8 (1.9)	34 (6.6)				
Every night	31 (7.5)	61 (11.8)				
General health			7.91	2	**0.019** ^ 3^	15
Poor/fair	131 (31.6)	207 (40.1)				
Good	129 (31.2)	152 (29.5)				
Very good/excellent	134 (37.2)	157 (30.4)				
Functional status			5.997	1	**0.014** ^ 1^	3
Independent	342 (81.4)	389 (74.5)				
Dependent on 1–5 activities	78 (18.6)	133 (25.5)				
Cognition			0.091	1	**0.763** ^ 1^	5
<24 MMSE	32 (7.6)	36 (6.9)				
≥24 MMSE	387 (92.4)	485 (93.1)				
Mood (below/over median = 1)			5.485	1	**0.019** ^ 1^	34
Low	209 (51.4)	299 (59.3)				
High	198 (48.6)	205 (40.7)				

*Note*: ^1^Yates' continuity correction, ^2^Mann-Whitney's *U*-test, and ^3^Pearson's *χ* ^2^ test.

**Table 3:** Leisure activities, age, gender, general health, functional status, mood, cognition, sleep duration, and sleep medication among good and poor sleepers in the total sample. Percentages are in brackets, significant values in bold.

	Good sleepers 0–3 sleep disturbances *n* = 664	Poor sleepers ≥4 sleep disturbances *n* = 220	*χ* ^2^ value	df	*P* value	Missing data (*n*)
Leisure activities						
Exercise	498 (76.9)	144 (67.3)	7.2	1	**0.007** ^ 2^	83
Gardening	491 (74.5)	125 (56.8)	23.8	1	**<0.001** ^ 2^	5
Strolling in the country	515 (78.1)	133 (60.5)	25.7	1	**<0.001** ^ 2^	5
Picking berries	373 (56.6)	89 (40.5)	16.6	1	**<0.001** ^ 2^	5
Hunting and fishing	117 (17.8)	17 (7.7)	12.1	1	**0.001** ^ 2^	5
Knitting, weaving, or sewing	202 (30.7)	89 (40.5)	6.7	1	**0.010** ^ 2^	5
Playing an instrument	93 (14.0)	24 (10.9)	1.1	1	0.286^2^	1
Painting, drawing, or pottery	59 (9.0)	18 (8.2)	0.0	1	0.125^2^	5
Home maintenance	376 (57.1)	71 (32.3)	39.5	1	<**0.001** ^2^	5
Repairing cars/machines	200 (30.3)	24 (10.9)	31.8	1	**<0.001** ^ 2^	5
Playing chess/cards	197 (29.7)	41 (18.6)	9.7	1	** 0.002** ^ 2^	1
Using/surfing the Internet/playing computer games	290 (43.7)	64 (29.1)	14.2	1	**<0.001** ^ 2^	1
Reading a daily paper	648 (97.7)	210 (95.5)	2.4	1	0.648^2^	1
Reading magazines/journals	573 (86.4)	186 (84.5)	0.3	1	0.56^2^	1
Reading books	485 (73.2)	164 (74.5)	0.1	1	0.751^2^	1
Watching TV	643 (97.0)	211 (95.9)	0.3	1	0.578^2^	1
Listening to music	593 (89.4)	193 (87.7)	0.3	1	0.562^2^	1
Age cohorts			29.5	2	**<0.001** ^ 1^	61
Retirement age (60- and 66-year-olds)	289 (43.5)	61 (27.7)				
72- and 78-year-olds	212 (31.9)	65 (29.5)				
≥81-year-olds	163 (24.5)	94 (42.7)				
Gender			45.9	1	**<0.001** ^ 2^	61
Women	322 (48.5)	165 (75.0)				
Men	342 (51.5)	55 (25.0)				
General health			91.9	2	**<0.001** ^ 1^	69
Poor/fair	180 (27.4)	131 (59.8)				
Good	202 (30.7)	62 (28.3)				
Very good/excellent	275 (41.9)	26 (11.9)				
Functional status			31.8	1	**<0.001** ^ 2^	64
Independent	551 (83.4)	144 (65.5)				
Dependent on 1–5 activities	110 (16.6)	76 (34.5)				
Mood (below/over median = 1)			31.0	1	**<0.001** ^ 2^	83
Low	315 (48.9)	154 (70.6)				
High	329 (51.1)	64 (29.4)				
Cognition			0.230	1	0.631^2^	66
<24 MMSE	43 (6.5)	17 (7.8)				
≥24 MMSE	617 (93.5)	202 (92.2)				
Sleep duration (hours)			114.8	2	**<0.001** ^ 1^	163
Short sleep ≤5 h	36 (6.2)	75 (36.6)				
Normal sleep 6–9 h	530 (91.9)	126 (61.5)				
Long sleep ≥10 h	11 (1.9)	4 (2.0)				
Prescribed sleep medication			217.6	4	**<0.001** ^ 1^	63
Never	578 (87.3)	93 (42.3)				
Sometimes per month	47 (7.1)	29 (13.2)				
Several times per month	4 (0.6)	7 (3.2)				
Sometimes per week	7 (1.1)	32 (14.5)				
Every night	26 (3.9)	59 (26.8)				

*Note*: ^1^Pearson's *χ* ^2^ test, ^2^Yates' continuity correction.

**Table 4:** The two clusters of activities associated with sleep disturbances, with and without adjustment for confounders.

	Model 1a			Model 1b		
	OR	95% CI	*P* value	OR	95% CI	*P* value
Physical outdoor activities			**0.007**			
None	2.15	0.92–5.01	0.077			
One	1.80	0.81–3.96	0.148			
Two	1.70	0.80–3.61	0.168			
Three	1.06	0.51–2.20	0.881			
Four	0.80	0.39–1.65	0.546			
Sociointellectual activities			**<0.001**			
None	3.75	1.49–9.43	**0.005**			
One	3.15	1.26–7.88	**0.014**			
Two	1.92	0.76–4.88	0.170			
Three	0.69	0.24–2.02	0.497			
Gender (women)				3.12	2.16–4.52	**<0.001**
Age cohorts						**0.011**
72- and 78-year-olds				0.93	0.60–1.44	0.742
81 years or older				1.68	1.09–2.58	**0.018**
General health						**<0.001**
Good				3.36	2.00–5.62	**<0.001**
Poor/fair				6.97	4.24–11.45	**<0.001**

Notes: in model 1a physical outdoor activities and sociointellectual activities were entered. The model explained between 8.9% (Cox and Snell *R* ^2^) and 13.2% (Nagelkerke *R* ^2^), Hosmer and Lemeshow 0.662, chi-square 5.870 (df 8), missing *n* = 85. Model 1b included physical outdoor activities and sociointellectual activities and was adjusted for gender, age, functional ability, mood, and general health. The model explained between 16.1% (Cox and Snell *R* ^2^) and 23.8% (Nagelkerke *R* ^2^), Hosmer and Lemeshow 0.497, chi-square 7.377 (df 8), missing *n* = 111. Significant factors are presented in bold. Only the last step of the regression analyses is shown in the table.

**Table 5:** Individual leisure activities associated with sleep disturbances, with and without adjusting for confounders.

	Model 2a			Model 2b		
	OR	95% CI	*P* value	OR	95% CI	*P* value
Gardening	1.45	1.00–2.08	**0.048**			
Strolling in the country	1.47	1.01–2.14	**0.046**			
Home maintenance	1.60	1.08–2.39	**0.020**			
Repairing cars/machines	2.20	1.28–3.77	**0.004**			
Playing chess/cards	1.69	1.13–2.52	**0.011**	1.54	1.01–2.35	**0.046**
Gender (women)				3.06	2.11–4.43	**<0.001**
Age cohorts						**0.019**
72- and 78-year-olds				0.91	0.59–1.41	0.666
81 years or older				1.60	1.04–2.47	**0.034**
General health						**<0.001**
Good				3.33	1.99–5.58	**<0.001**
Poor/fair				6.82	4.14–11.22	**<0.001**

Note: in Model 2a exercise, gardening, strolling in the country, picking berries, hunting/fishing, home maintenance, repairing cars/machines, knitting/weaving/sewing, playing chess/cards, and using/surfing the Internet/playing computer games were entered. The model explained 8.2% (Cox and Snell *R* ^2^) to 12.1% (Nagelkerke *R* ^2^) of the variance, Hosmer and Lemeshow 0.563, chi-square 6.670 (df 8), missing *n* = 85. Model 2b included exercise, gardening, strolling in the country, picking berries, hunting/fishing, home maintenance, repairing cars/machines, knitting/weaving/sewing, playing chess/cards, and using/surfing the Internet/playing computer games and was adjusted for gender, functional ability, mood, general health, and age. The model explained between 16.5% (Cox and Snell *R* ^2^) and 24.4% (Nagelkerke *R* ^2^) of the variance, Hosmer and Lemeshow 0.260, chi-square 10.078 (df 8), missing *n* = 111. Only the last step of the regression analyses is shown in the table.

**Table 6:** Individual leisure activities and gender interactions associated with sleep disturbances, with and without adjusting for confounders.

	Model 3a			Model 3b		
	OR	95% CI	*P* value	OR	95% CI	*P* value
Gardening	1.50	1.04–2.16	**0.032**			
Strolling in the country	1.56	1.07–2.29	**0.022**			
Playing chess/cards	1.61	1.09–2.39	**0.017**	1.43	0.95–2.17	0.090
Home maintenance	2.26	1.17–4.35	**0.015**	2.09	1.07–4.07	**0.031**
Home maintenance × gender	0.44	0.20–0.94	**0.033**	0.33	0.15–0.75	**0.008**
Gender (women)	3.63	2.15–6.15	**<0.001**	4.86	2.75–8.61	**<0.001**
Age cohorts						**0.043**
72- and 78-year-olds				0.95	0.61–1.49	0.832
81 years or older				1.56	0.99–2.46	0.056
General health						**<0.001**
Good				3.40	2.02–5.73	**<0.001**
Poor/fair				7.50	4.27–11.81	**<0.001**

Note: in Model 3a gardening, strolling in the country, home maintenance, repair cars/machines, and playing chess/cards were entered together with gender and the interactions gardening × gender, strolling in the country × gender, playing chess/cards × gender, repairing cars/machines × gender, and home maintenance × gender. The model explained between 9.2% (Cox and Snell *R* ^2^) and 13.7% (Nagelkerke *R* ^2^) of the variance, Hosmer and Lemeshow 0.887, chi-square 2.973, (df 7), missing *n* = 66. Model 3b included gardening, strolling in the country, home maintenance, repair cars/machines, and playing chess/cards, gender, gardening × gender, strolling in the country × gender, playing chess/cards × gender, repairing cars/machines × gender, and home maintenance × gender and was adjusted for age, functional ability, mood, and general health. The model explained between 16.9% (Cox and Snell *R* ^2^) and 24.9% (Nagelkerke *R* ^2^) of the variance, Hosmer and Lemeshow 0.716, chi-square 5.386, (df 8), missing = 93. Only the last step of the regression analyses is shown in the table.
